# Feasibility study on heterotrophic utilization of galactose by *Chlorella sorokiniana* and promotion of galactose utilization through mixed carbon sources culture

**DOI:** 10.1186/s13068-024-02547-9

**Published:** 2024-07-16

**Authors:** Shengjie Wu, Xiao Cheng, Qinyun Xu, Shikai Wang

**Affiliations:** College of Bioscience and Biotechnology, Yangzhou University, Yangzhou, 225009 People’s Republic of China

**Keywords:** *Chlorella sorokiniana*, Heterotrophic, Galactose, Mixed carbon sources, Biomass

## Abstract

**Background:**

The development of alternative carbon sources is important for reducing the cost of heterotrophic microalgae cultivation. Among cheap feedstocks, galactose is one of the most abundant sugars and can be easily obtained from many natural biomasses. However, it is generally difficult to be utilized by microalgae. In addition, the mechanism of its low utilization efficiency in heterotrophic cultivation is still unknown.

**Results:**

Among seven tested carbon sources, only glucose and acetate could be efficiently utilized by *C. sorokiniana* in heterotrophic cultivation while there were no apparent signs of utilization of other carbohydrates, including galactose, in regular heterotrophic cultivation. However, galactose could be utilized in cultures with high inoculation sizes. This confirmed that *C. sorokiniana* has a complete pathway for transporting and assimilating galactose under dark conditions, but the rate of galactose utilization is quite low. In addition, the galactose utilization was greatly enhanced in mixotrophic cultures, which indicated that galactose utilization could be enhanced by additional pathways that can enhance cell growth. Based on above results, a mixed carbon source culture strategy was proposed to improve the utilization rate of galactose, and a significant synergistic effect on cell growth was achieved in cultures using a mixture of galactose and acetate.

**Conclusions:**

This study indicated that the galactose metabolism pathway may not be inherently deficient in Chlorophyta. However, its utilization rate was too low to be detected in regular heterotrophic cultivation. Mixed carbon source culture strategy was confirmed effective to improve the utilization rate of galactose. This study contributes to a deeper understanding of the utilization ability of difficultly utilized substrates in the heterotrophic cultivation of microalgae, which is of great significance for reducing the cost of heterotrophic cultivation of microalgae.

## Background

Microalgae have attracted increasing attention in recent years due to their promising potential applications in food, energy, and high-value compound production, as well as in environmental fields [1]. The abundant production of algal biomass is necessary to support its wide application. Microalgae can grow via various trophic modes. Among them, autotrophic and heterotrophic cultivation are the main approaches used in the commercial production of microalgae [2]. Compared to autotrophy, heterotrophic cultivation can avoid light dependence and greatly enhance the cell growth rate. It was estimated that the biomass productivity of heterotrophic cultures can exceed that of autotrophic cultures by one order of magnitude [2]. In addition, the design, operation, and scale-up of bioreactors are also greatly simplified in heterotrophic cultures [3]. Although no consensus has been reached on which is the most economical strategy by specialists, heterotrophic cultivation has been widely recognized as an important approach for bulk microalgal biomass production, especially for high-quality biomass production [4].

From an economic point of view, the cost of organic carbon sources is one of the major costs for heterotrophic cultivation [5]. Currently, glucose and glucose-rich substrates are the most commonly used carbon sources in heterotrophic microalgae cultivation due to their excellent culture performance compared to that of any other substrate [6, 7]. In addition to glucose, acetate and glycerol are also frequently used in the cultivation of specific species, such as *Haematococcus pluvialis* and *Schizochytrium* sp., respectively [8, 9]. Some natural organic substrates or wastes, such as sucrose-rich molasses, lactose-rich cheese whey, xylose and galactose-rich hemicellulose, are promising alternative carbon sources for the heterotrophic cultivation of microalgae because they are abundant, inexpensive and renewable. Considering that disaccharides and polysaccharides, such as sucrose and starch, are difficult for microalgae to directly utilize [3, 10], heterotrophic cultivation of microalgae using corresponding monomer substances is of great significance for reducing carbon source costs.

Among cheap feedstocks, galactose is one of the most abundant sugars and can be easily obtained from agar and cellulosic components of red seaweed, hemicellulose from lignocellulose, and lactose from dairy waste. It usually exists as a mixture with glucose [11, 12]. However, galactose is difficult for most Chlorophyta to directly utilize in heterotrophic cultures and even in mixotrophic modes [13, 14]. Although galactose is an epimer of glucose and only the orientation of the hydroxyl group on the fourth carbon is different from that of glucose, the metabolic pathway for galactose assimilation is more complicated than that for glucose [15]. In most organisms, galactose is metabolized via the conserved Leloir pathway [16]. It was hypothesized that the disruption of key biosynthetic or degradative pathways was a primary cause of the inability of cells to utilize certain carbon sources [17]. It has been found that some Chlorophyta are capable of assimilating galactose under light conditions [18, 19]. This indicated that the galactose metabolism pathway may not be inherently deficient in Chlorophyta. However, the specific mechanism underlying the poor utilization efficiency of galactose in the heterotrophic cultivation of *Chlorella* and other Chlorophyta remains unknown.

Previous studies have indicated that mixed carbon sources are favorable for cell growth or product accumulation rather than being the sole carbon source, for many microalgal species [20–22]. Whangchai et al. [23] reported that the synergistic supplementation of organic carbon substrates could increase the quality of the products. In addition, Huang et al. [24] reported that the combination of acetic acid and glucose as a carbon source could maintain the pH during the culture period and significantly enhance the biomass production of *Chlorella*. Hawkins [25] reported that xylose is difficult for *Chlorella* to utilize when it was used alone. However, it could be fully utilized when added to the culture combined with glucose. This indicated that the synergistic supplementation of carbon sources might improve the utilization rate of some difficultly utilized substrates.

In this study, the heterotrophic cultivation of a newly isolated *C. sorokiniana* was first investigated using different carbon sources. Subsequently, considering that the utilization of galactose may be neglected due to its low utilization rate in heterotrophic cultures with general low inoculation sizes, heterotrophic culture using galactose with different inoculation sizes was tested to explore the feasibility of heterotrophic galactose utilization by *C. sorokiniana*. To further explore the relationship between the cell growth and galactose utilization*,* galactose, which is difficult to be utilized in general heterotrophic cultivation, was tested for its utilization effect in mixotrophic cultivation. A possible explanation for the increased utilization rate of galactose by *C. sorokiniana* was proposed according to the above experimental results. Finally, galactose was mixed with easily utilized carbohydrates to explore effective approaches to improve its utilization efficiency. This study provides confidence in the feasibility of heterotrophic cultivation of microalgae using carbon sources that are commonly considered difficult to utilize. In addition, it provides an effective strategy for improving the culture performance of difficultly utilized carbon sources.

## Materials and methods

### Microalgae strains and inoculum culture

*C. sorokiniana* (CCTCC M 20221125) was isolated from a local river which is contaminated by organic waste and stored in our laboratory. For inoculum culture, a single colony on solid BG-11 medium [26] was inoculated in liquid BG-11 medium supplemented with 5 g/L glucose and incubated at 30 °C in an orbital shaker at 150 rpm. After heterotrophic culture for 3 days, the cells were collected by centrifugation at 6796 × *g* for 5 min. Then, the cells were washed three times with deionized water and used as inoculum for subsequent experiments.

### Experimental design

#### Heterotrophic culture with single-carbon sources

To investigate the ability to heterotrophically utilize different carbon sources of *C. sorokiniana*, BG-11 medium individually supplemented with 5 g/L of seven common carbon sources (glucose, galactose, fructose, xylose, sucrose, lactose, and sodium acetate) was used as the medium. Subsequently, to investigate the effect of inoculation sizes on the utilization of galactose, BG-11 medium supplemented with 3 g/L galactose was used as the medium and the initial inoculation sizes were set as 0.012, 0.25, 0.50, 0.75, 1.00, and 1.25 g/L.

#### Mixotrophic culture with galactose

BG-11 medium supplemented with 5 g/L galactose was used for the mixotrophic culture at the light intensities of 30 and 60 μmol m^−2^ s^−1^. In addition, a photosynthetic block experiment involving the mixotrophic cultivation of *C. sorokiniana* was performed in BG-11 medium supplemented with 3 g/L galactose. Dichlorophenyl dimethyl urea (DCMU) was purchased from RHAWN (Shanghai, China) and dissolved in dimethyl sulfoxide (DMSO). After being filtered through a 0.22 μm sterilized membrane, the solution was added to the culture at a concentration of 8 μM [27]. Cultures treated with an equal volume of DMSO were used as controls.

#### Mixed carbon sources culture

A mixed carbon source strategy was tested for its ability to promote galactose utilization in the heterotrophic cultivation of *C. sorokiniana*. Galactose was used with easily utilized carbon sources, including glucose and acetate. Considering the molecular weight of acetate is different with galactose, the mixing ratio is represented by carbon ratio and maintained at a constant galactose concentration of 2.5 g/L (Table 1).

**Table 1:** The carbon source dosage in heterotrophic cultivation experiments using mixed carbon sources

Carbon ratio of glucose/acetate to galactose	Combination of galactose and glucose		Combination of galactose and acetate	
	Glucose dosage (g/L)	Galactose dosage (g/L)	Sodium acetate dosage (g/L)	Galactose dosage (g/L)
0	0	2.50	0	2.50
0.2	0.50	2.50	0.70	2.50
0.4	1.00	2.50	1.35	2.50
0.6	1.50	2.50	2.05	2.50
0.8	2.00	2.50	2.75	2.50
1.0	2.50	2.50	3.40	2.50

#### Culture conditions

Except for the experiment on heterotrophic culture using galactose with different inoculation sizes, the initial inoculation size was 0.012 g/L. All cultures were performed in 250 mL Erlenmeyer flasks with 100 mL of medium and incubated at 30 °C in an orbital shaker at 150 rpm.

### Analytic methods

#### Determination of cell growth

The biomass concentration (X, g/L) during the culture period was spectrophotometrically determined by measuring the optical density at 680 nm (OD_680 nm_) and calculated according to the calibration curve of biomass concentration and optical density [28]. The calibration curve for *C. sorokiniana* was shown as Eq. (1):

1 $${\text{X}} = 0.{3267}*{\text{OD}}_{{{68}0{\text{ nm}}}} - 0.0{5}0{9 }\left( {{\text{R}}^{{2}} = 0.{998}} \right)$$

As acetate was added in the form of sodium acetate, the conversion ratio of carbon source to biomass was defined as the weight of biomass accumulated by consumption per mole-C equivalent of carbon source, and Rc (g-biomass/mol-C) was calculated as Eq. (2):

2 $${\text{R}}_{{\text{c}}} = \Delta {\text{C}}_{{\text{m}}} /\Delta {\text{C}}_{{\text{c}}}$$

where ∆C_m_ is the increasing algal biomass (g/L) and ∆C_c_ is the carbon molar concentration of the consumed carbon source (mol/L).

#### Determination of the carbon source concentration

The culture suspension was first centrifuged at 4 °C and 6796 × *g* for 10 min, and then filtered through a 0.22 μm membrane. The sample was analyzed using an HPLC instrument (Agilent 1260, America) equipped with a Bio-Rad Aminex^®^ HPX-87H column (300 mm *L* × 7.8 nm *I.D.*) with an injection volume of 20 μL. The RID was used as the detector for the determination of glucose, galactose, fructose, xylose, sucrose, and lactose, and the detector temperature was set at 40 °C. VWD was used as the detector for the determination of acetate and the wavelength was set at 210 nm. The column temperature was set at 60 °C and 5 mM H_2_SO_4_ was used as the mobile phase with a flow rate of 0.5 mL/min for all measurements.

#### Determination of lipid, protein, and starch contents

The cells were first collected by centrifugation at 4 °C and 6796 × *g* for 10 min. Then, the cells were lyophilized using a lyophilizer (FD-1A-50, BiLon, China). The lipid and protein contents were analyzed using organic solvent extraction method and potassium persulfate oxidation method according to the methods reported by Wang et al. [3] and Tian et al. [29], respectively. For the determination of starch content, the lyophilized cells were first ground for 10 min using a mortar and pestle. Then, it was measured by visible spectrophotometry method using a starch detection kit (BC0700, Solarbio, China) according to the manufacturer’s instructions.

### Statistical analysis

The data are presented as the means ± standard errors of the means of three parallel experiments. The statistical significance was analyzed by one-way analysis of variance (ANOVA) (P < 0.05) using Origin 9 (OriginLab, USA).

## Results and discussion

### Heterotrophic culture of *C. sorokiniana* using different carbon sources

**Fig. 1:**
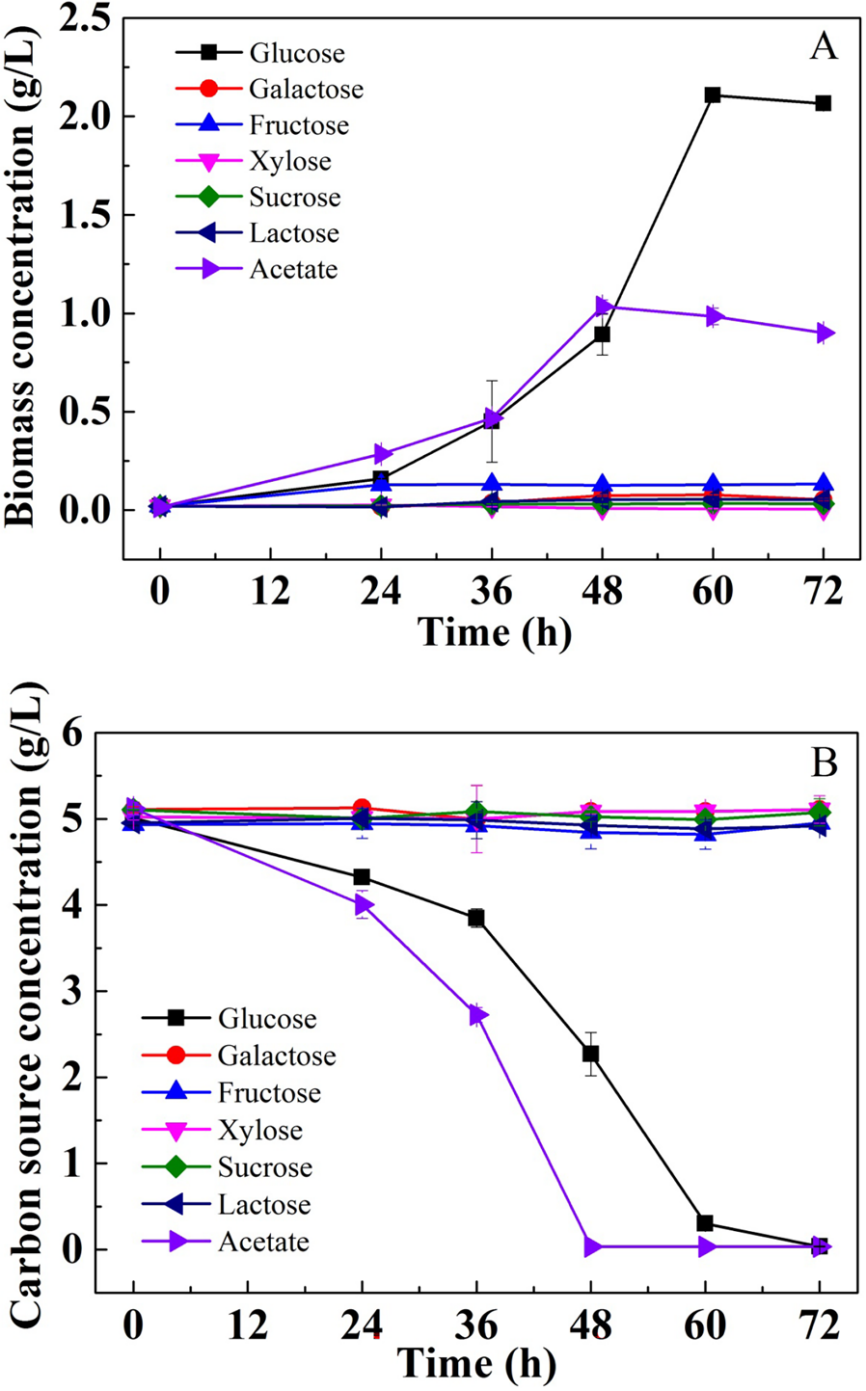
Cell growth (**A**) and carbon source consumption (**B**) in the heterotrophic cultivation of *C. sorokiniana* using different carbon sources

Similar to the results found in most studies, glucose exhibited the best culture performance for the newly isolated *C. sorokiniana* (Fig. 1A). The biomass concentration reached 2.11 g/L after being cultured for 60 h. Meanwhile, the glucose was exhausted after 72 h (Fig. 1B). The conversion ratio of glucose to biomass reached 13.31 g-biomass/mol-C (Table 2). Acetate was also capable of supporting the rapid growth of *C. sorokiniana*, but it resulted in a significant lower (P < 0.05) biomass yield than glucose, as the highest biomass concentration was only 1.01 g/L. The conversion ratio of acetate to biomass was only 8.22 g-biomass/mol-C, which was only 62% of that of glucose to biomass (Table 2). However, *C. sorokiniana* was unable to heterotrophically utilize other sugars, including galactose, fructose, xylose, sucrose, and lactose, as negligible cell growth and sugar consumption were observed during the culture period (Fig. 1A, B).

**Table 2:** The conversion ratio of carbon source to biomass in the heterotrophic cultivation of *C. sorokiniana* using different carbon sources

Carbon source	Galactose consumption (mol-C/L)	Glucose consumption (mol-C/L)	Acetate consumption (mol-C/L)	Biomass accumulation (g/L)	Rc (g-biomass/mol-C)
5 g/L Glucose^#^	N.A	0.157 ± 0.002	N.A	2.09 ± 0.03	13.31 ± 0.08a
5 g/L Acetate^##^	N.A	N.A	0.124 ± 0.002	1.02 ± 0.03	8.22 ± 0.07d
3 g/L Galactose^##^	0.094 ± 0.002	N.A	N.A	0.72 ± 0.02	7.66 ± 0.05e
2.5 g/L Galactose + 2.5 g/L Glucose^###^	0.021 ± 0.001	0.084 ± 0	N.A	1.32 ± 0.07	12.57 ± 0.1b
2.5 g/L Galactose + 3.4 g/L Acetate^###^	0.077 ± 0.002	N.A	0.065 ± 0	1.44 ± 0.02	10.14 ± 0.08c

Values in a column with different letters are significantly different according to one-way analysis of variance (ANOVA) (P < 0.05) and the values decreased sequentially from letter a to e

^#^The data were calculated from Fig. 1

^##^The data were calculated from Fig. 3

^###^The data were calculated from Fig. 5; N.A.: not available

Glucose has been widely confirmed as the optimal organic carbon source for the cultivation of most heterotrophic algal species due to its higher energy content per mole compared with that of other substrates [6]. Many eukaryotic microalgae can also take up acetate using monocarboxylic/proton transporter proteins and oxidize it through the glyoxylate cycle or the tricarboxylic acid cycle [6]. In addition, the utilization rate of acetate was even greater than that of glucose. Xie et al. [30] reported that acetate was preferentially utilized when glucose and acetate were simultaneously used for the culture of *C. saccharophila*. It is widely recognized that disaccharides usually result in negative outcomes in terms of cell growth and metabolite production. This might be due to the deficiency of disaccharide carriers in most microalgae [18, 31]. Surprisingly, the culture performance of the tested monosaccharides was also poor, except for that of glucose. For the utilization of organic carbon sources by microalgae, they should first be transported across the plasmalemma by a specific transport system and then be assimilated by the central carbon metabolism system. The hexose uptake protein-encoding gene (*hup*) of *Chlorella* has been cloned, and its functional mechanism has also been characterized [32–34]. In general, *Chlorella* possesses three hexose transporter genes (*hup1*, *hup2* and *hup3*). They can all be induced by d-glucose. In addition, they are also capable of transporting substrates other than glucose. For example, HUP1 and HUP3 can efficiently transport d-fructose and d-mannose, while HUP2 can efficiently transport d-galactose and d-xylose [35]. *C. sorokiniana* induced by d-glucose exhibited a markedly increased d-xylose uptake rate compared with uninduced cells [36]. Therefore, the inability to utilize galactose and fructose should not be due to the lack of a transport system, as glucose can be efficiently utilized by *C. sorokiniana*. It has been proposed that the inefficient utilization of galactose and fructose might be due to incomplete pathways or the absence of an enzymatic reaction in central carbon metabolism [31]. The detailed mechanism needs to be further clarified.

### Heterotrophic culture of *C. sorokiniana* using galactose at different inoculation sizes

**Fig. 2:**
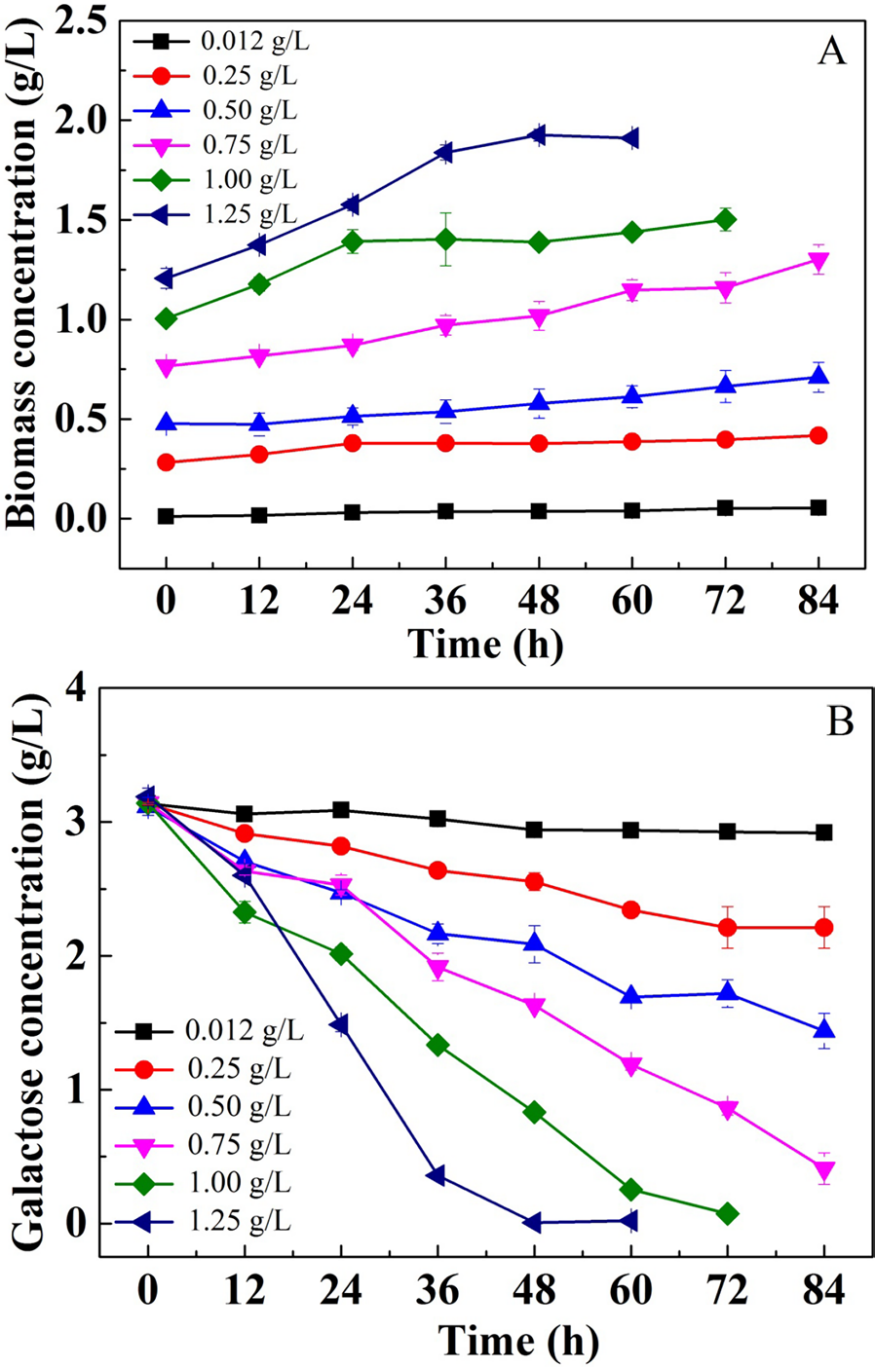
Heterotrophic cultivation of *C. sorokiniana* using galactose with different inoculation sizes (**A** cell growth curve; **B** galactose consumption curve)

As shown in Fig. 2A, although negligible cell growth was observed in cultures with a low inoculation concentration of 0.012 g/L, obvious cell growth was observed in cultures with greater inoculation concentrations. In addition, the biomass productivity increased with increasing inoculation size. They reached 0.51, 1.61, 2.78, 6.39, 16.11, and 17.53 mg L^−1^ h^−1^ in the cultures with inoculation rates of 0.012, 0.25, 0.50, 0.75, 1.00, and 1.25 g/L, respectively. In accordance with cell growth, the consumption rate of galactose was also positively correlated with inoculation size (Fig. 2B). Galactose was difficult to be utilized in cultures with a low inoculation concentration of 0.012 g/L. Increasing the inoculation size significantly promoted the utilization of galactose. In the cultures with inoculation concentrations of 0.012, 0.25, 0.50, 0.75, 1.00, and 1.25 g/L, the galactose consumption rates reached 2.59, 11.00, 19.97, 32.52, 48.12, and 78.62 mg L^−1^ h^−1^, respectively. In addition, galactose could be exhausted after being cultured for 72 and 48 h with inoculation concentrations of 1.00 and 1.25 g/L, respectively. The above results confirmed that *C. sorokiniana* has a complete pathway for transporting and assimilating galactose under dark conditions.

In general, the inoculation size in microalgae cultivation is relatively low and the initial OD at 680 nm is usually no more than 0.5. Therefore, the effect of inoculation size on the utilization efficiency of substrates has seldom been investigated. Surprisingly, galactose could be efficiently utilized by *C. sorokiniana* in cultures with high inoculation sizes. This confirmed that *C. sorokiniana* has a complete pathway for transporting and assimilating galactose under dark conditions. Although the galactose utilization rate of cell is low, it could be significantly enhanced by the increase in the cell population. Therefore, effective approaches to promote cell growth are necessary to improve the efficiency of galactose utilization.

### Mixotrophic cultivation of *C. sorokiniana* using galactose

**Fig. 3:**
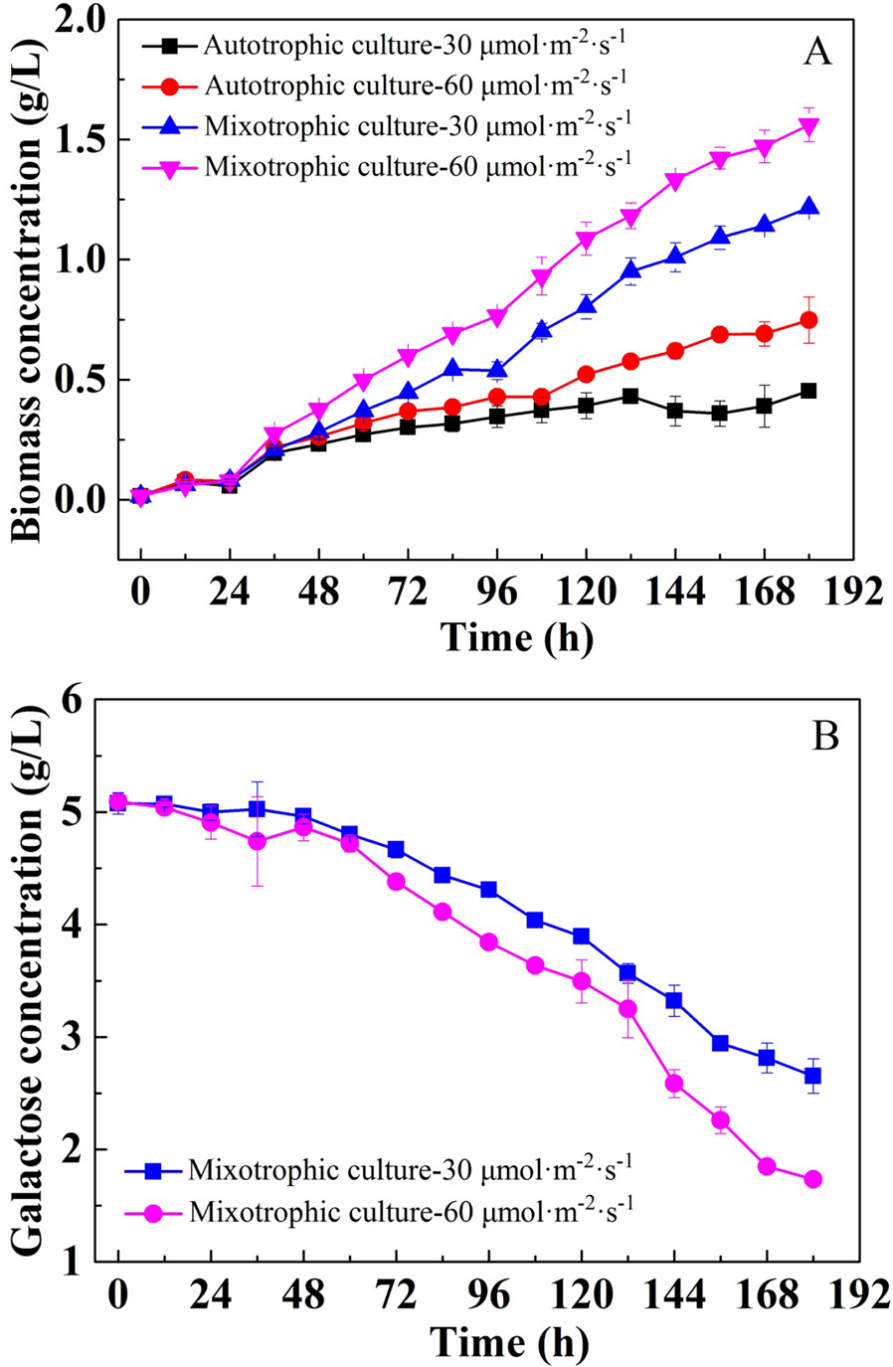
Mixotrophic cultivation using 5 g/L galactose and photoautotrophic cultivation of *C. sorokiniana* under different light intensities (**A** cell growth curve; **B** galactose consumption curve)

*C. sorokiniana* can inherently utilize galactose under dark conditions at a relatively low rate. However, galactose assimilation was significantly enhanced when the cells were cultured in mixotrophic mode. As shown in Fig. 3, galactose was continuously consumed, and the addition of galactose significantly enhanced the growth rate of *C. sorokiniana* compared with that of autotrophic cultures (P < 0.05). In addition, in line with the trend observed in autotrophic culture, the cell growth rate in mixotrophic cultures also increased with increasing light intensity as the specific growth rate increased from 0.024 to 0.026 h^−1^ as the light intensity increased from 30 to 60 μmol m^−2^ s^−1^. As shown in Fig. 3B, in the early stages of cultivation, the consumption of galactose was almost imperceptible, especially in the cultures under a light intensity of 30 μmol m^−2^ s^−1^. Afterwards, galactose was rapidly consumed during the logarithmic growth phase, and the galactose uptake rate followed the same trend as that of cell growth. The galactose consumption rate reached 18.67 mg L^−1^ h^−1^ in cultures under a light intensity of 60 μmol m^−2^ s^−1^, which is significant higher (P < 0.05) than that of cultures under a light intensity of 30 μmol m^−2^ s^−1^ (13.47 mg L^−1^ h^−1^). After being cultured for 180 h, the residual galactose concentration was only 1.73 g/L in cultures exposed to 60 μmol m^−2^ s^−1^ of illumination, whereas in cultures grown under 30 μmol m^−2^ s^−1^, it was still as high as 2.65 g/L. This indicated that light could increase the utilization rate of galactose by *C. sorokiniana*.

Similar phenomena were also found in other algal species. Subramanian et al. [37] reported that light was necessary for *C. vulgaris* to assimilate glucose, as it did not grow in glucose-sufficient but dark conditions. In addition, *Asterarcys* sp. SCS-1881, *Scenedesmus quadricauda*, and *S. obliquus* were incapable of utilizing glucose, xylose, and lactose under dark conditions, respectively. However, these substrates can be efficiently utilized when the cells are transferred under light conditions [38–40]. One possible explanation for this phenomenon is that light can enhance the expression levels of sugar transporters or that the sugar transport process requires additional energy, which is provided by a light-dependent reaction [36]. In addition, the catabolism of these substrates under dark conditions requires additional energy, cofactors or other metabolic pathways that could be obtained from light metabolism [18, 25]. However, the detailed mechanism of utilization distinction of sugars in heterotrophic and mixotrophic cultures is still unclear.

**Fig. 4:**
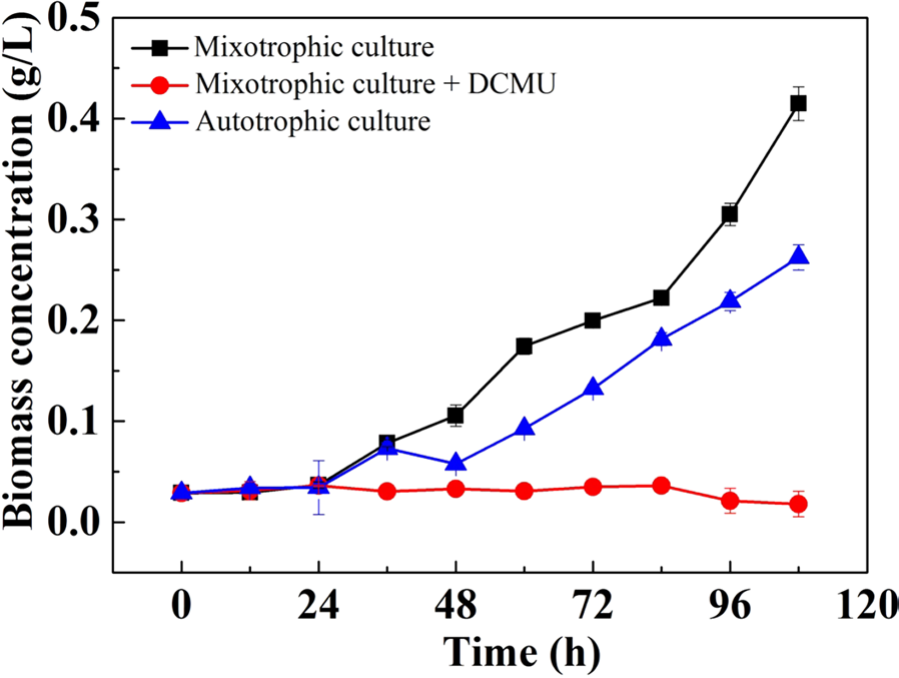
Influence of DCMU addition on the mixotrophic cultivation of *C. sorokiniana* using galactose

For photosynthetic autotrophic microorganisms, light not only provides energy to cells, but also serves as an important inducer that is capable of regulating the expression of many metabolic pathways. Considering that galactose could be efficiently utilized under light conditions but not under dark conditions, it is speculated that light may stimulate the expression of genes related to galactose metabolism pathways. However, the results of this study refute this speculation. As shown in Fig. 4, the assimilation of galactose was completely inhibited by blocking the photoreaction of photosynthesis with the addition of DCMU under light conditions. DCMU is an herbicide that can specifically block electron flow from photosystem II, thereby inhibiting NADPH production [18]. This indicated that light could not directly affect the utilization of galactose. The promotion of the utilization efficiency of galactose by light was mainly based on the energy generated by photoreactions. The effect of energy on the utilization rate of galactose may be twofold. On the one hand, the utilization rate of galactose could be promoted by the increasing cell population, which was caused by the energy generated through photoreactions; on the other hand, the assimilation of galactose relies on the energy provided by photoreactions. Based on the results shown in Figs. 2 and 3, the enhanced utilization of galactose in mixotrophic cultures was more likely related to the cell growth stimulated by photosynthesis. Therefore, apparent galactose utilization could be observed as the utilization rate of galactose drastically increased with the increasing cell population.

Based on the above results, it could be concluded that *C. sorokiniana* has a complete pathway for transporting and assimilating galactose under dark conditions. However, the utilization rate of galactose in heterotrophic cultures is quite low. Galactose is generally metabolized via the Leloir pathway in most organisms. After multiple enzymatic reactions, galactose is ultimately converted into glucose-6-phosphate, which can enter different metabolic pathways [16]. The low utilization rate of galactose may be constrained by the Leloir pathway. It is feasible to improve its utilization rate by overexpressing related rate-limiting enzyme-encoding genes, such as UTP-Gal-1P Uridylyl-transferase and UDP-Gal 4-Epimerase, in the Leloir pathway [41]. In addition, the galactose utilization efficiency could also be enhanced by additional pathways that can promote cell growth.

### Promotion of the heterotrophic utilization of galactose by *C. sorokiniana* through a mixed carbon sources culture strategy

**Fig. 5:**
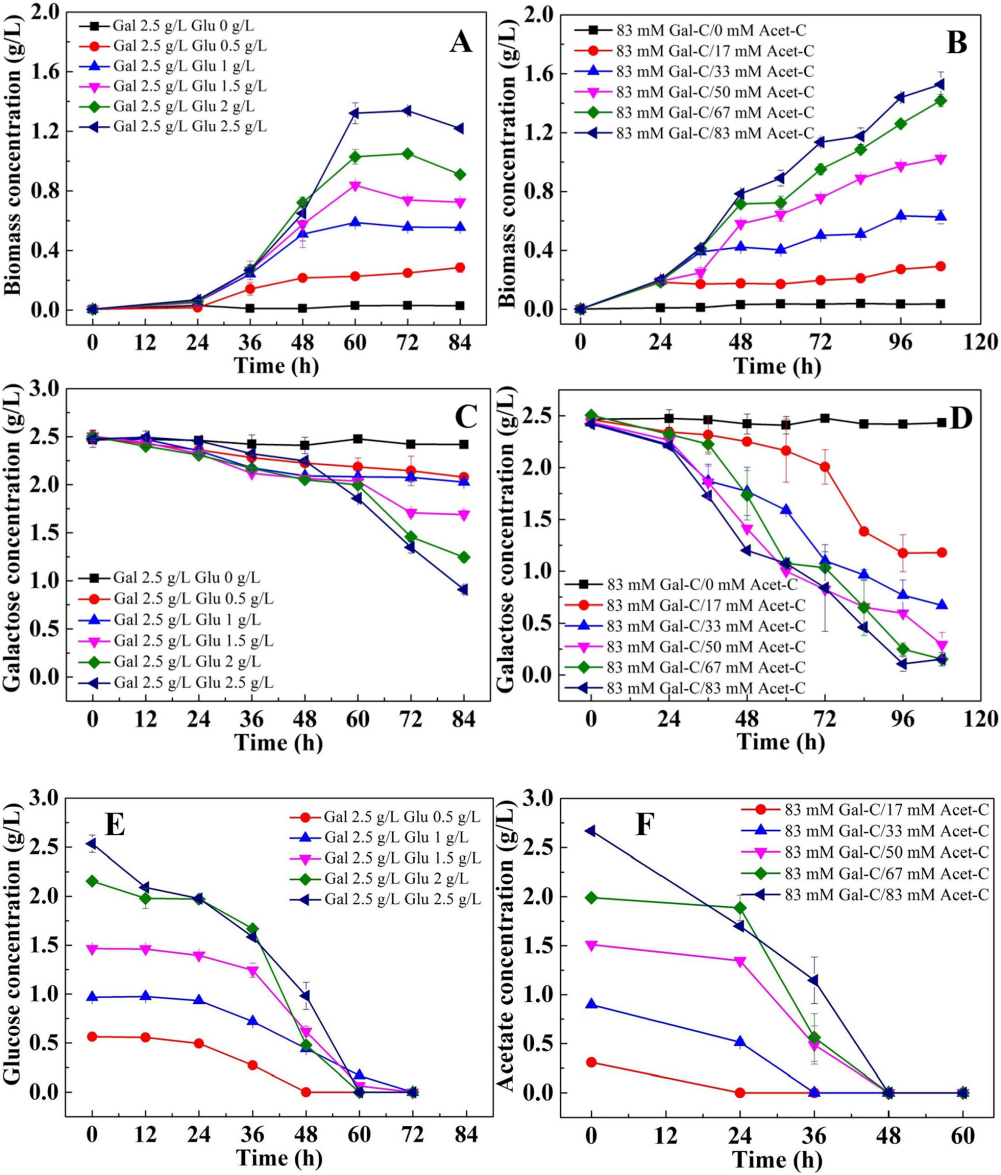
Cell growth and carbon source consumption in the heterotrophic cultivation of *C. sorokiniana* using mixed carbon sources. **A**, **B** cell growth curve; **C**, **D** galactose consumption curve; **E**, **F** glucose and acetate consumption curve, respectively. **A**, **C**, **E** Cultures using galactose mixed with different ratios glucose; **B**, **D**, **F** cultures using galactose mixed with different ratios acetate

As shown in Fig. 5A, B, increasing either glucose or acetate addition greatly enhanced cell growth. In addition, the biomass yield in cultures with mixed carbon sources was significantly greater (P < 0.05) than that in cultures with the corresponding amount of glucose or acetate alone. The highest biomass concentrations reached 0.23, 0.59, 0.84, 1.03, and 1.32 g/L in the cultures using 2.5 g/L galactose mixed with 0.5, 1.0, 1.5, 2.0, and 2.5 g/L glucose, respectively. In comparison, the final biomass was only 0.10, 0.34, 0.57, 0.68, and 0.94 g/L in cultures with the corresponding amounts of glucose. Similar results were also found in cultures using acetate (Fig. 5B). This indicated that the addition of easily utilized carbon sources, e.g., glucose and acetate, could promote the utilization of difficultly utilized carbon sources. This could be further confirmed by the results of galactose consumption. As shown in Fig. 5C, D, with the addition of glucose or acetate, galactose could be efficiently utilized by *C. sorokiniana*. In addition, the rate of galactose utilization was positively correlated with the amount of added glucose or acetate. The galactose consumption rates reached 5.04, 5.47, 9.72, 14.96, and 18.68 mg L^−1^ h^−1^ in the cultures using 2.5 g/L galactose mixed with 0.5, 1.0, 1.5, 2.0, and 2.5 g/L glucose, respectively (Fig. 5C). In addition, acetate promoted the utilization of galactose better than glucose, as the galactose consumption rates reached 13.43, 17.34, 19.21, 23.50, and 24.11 mg L^−1^ h^−1^ in the cultures with carbon element ratios of acetate and galactose of 0.2, 0.4, 0.6, 0.8, and 1.0, respectively (Fig. 5D). This might be related to the greater rate of acetate utilization by *C. sorokiniana*. As shown in Fig. 5E, F, acetate was exhausted at 48 h in all cultures, while glucose was not completely consumed until 72 h in most of the cultures. The combination of galactose and acetate exhibited a significant synergistic effect on cell growth promotion. As shown in Table 2, the biomass conversion ratio reached 10.14 g-biomass/mol-C in cultures using a mixture of acetate and galactose, while that in cultures using galactose and acetate alone was only 7.66 and 8.22 g-biomass/mol-C, respectively (Table 2). However, no synergistic effect on cell growth promotion was observed in the cultures using the mixture of galactose and glucose, as the biomass conversion ratio was only 12.57 g-biomass/mol-C, which is lower than that in cultures using glucose alone (Table 2). In addition, no carbon catabolite repression effect for galactose was observed in the cultures mixed with either glucose or acetate, as the utilization of galactose occurred simultaneously with that of glucose and acetate.

Glucose and acetate are the most commonly used carbon sources for heterotrophic culture of many algal species. The cell growth rate of heterotrophic cultures using galactose could be significantly enhanced by the assimilation of these easily used carbon sources. This study confirmed that *C. sorokiniana* could slowly utilize galactose. The increase in the cell growth rate achieved through the assimilation of glucose or acetate could synchronously improve the utilization rate of galactose. A similar phenomenon was also reported by Hawkins [25], who reported that the utilization rate of xylose could be greatly enhanced by the addition of glucose to *Chlorella*. This might be attributed to the same mechanism as in this study. Mixing galactose with other easily used carbon sources was an effective strategy for improving its utilization efficiency. In addition, acetate was a better choice than glucose, as a significant synergistic effect on cell growth promotion was achieved in the cultures using a mixture of galactose and acetate. This synergistic effect might be due to the pH balance caused by acetate and galactose metabolism [30]. The combination of difficultly used and easily used carbon sources was presented as a promising strategy for improving the utilization efficiency of some difficultly used substrates. This approach could not only increase the utilization rate of difficultly utilized carbon sources, but also significantly enhance the biomass conversion ratio of easily used carbon sources. Considering that many natural biomasses exist in the form of mixed sugars, this strategy is helpful for the exploitation of the hydrolysates of natural biomass to cultivate microalgae, which is beneficial for reducing the cultivation cost of microalgae [42].

## Conclusions

Many carbon sources, including galactose, are often considered difficult for microalgae to utilize via heterotrophic methods. This study confirmed that *C. sorokiniana* is capable of heterotrophically utilizing galactose. However, the utilization of galactose is generally neglected due to its very low utilization rate in general cultures. Based on the finding that the utilization of galactose can be enhanced through additional strategies aimed at promoting cell growth, such as the employment of mixotrophic cultures, a strategy of mixing galactose with easily utilized carbon sources was proposed and proven effective. This approach not only significantly increased the assimilation rate of galactose but also exhibited a synergistic effect on cell growth. This mixed carbon source strategy could be used to exploit alternative carbon sources in the heterotrophic cultivation of microalgae as many natural biomasses exist in the form of mixed sugars. This study contributes to a deeper understanding of the utilization ability of difficultly utilized substrates in the heterotrophic cultivation of microalgae, which is helpful for exploring the mechanism of other carbon sources assimilation in microalgae and reducing the cost of heterotrophic microalgae cultivation.

## Data Availability

Data will be made available on request.
